# In Vitro Bacterial Competition of *Staphylococcus aureus*, *Streptococcus agalactiae*, and *Escherichia coli* against Coagulase-Negative Staphylococci from Bovine Mastitis Milk

**DOI:** 10.3390/antibiotics12030600

**Published:** 2023-03-16

**Authors:** Anyaphat Srithanasuwan, Montira Intanon, Wasana Chaisri, Witaya Suriyasathaporn

**Affiliations:** 1 Department of Food Animal Clinic, Faculty of Veterinary Medicine, Chiang Mai University, Chiang Mai 50100, Thailand; 2 Department of Veterinary Bioscience and Veterinary Public Health, Faculty of Veterinary Medicine, Chiang Mai University, Chiang Mai 50100, Thailand; 3 Research Center of Producing and Development of Products and Innovations for Animal Health and Production, Chiang Mai University, Chiang Mai 50100, Thailand; 4 Cambodia Campus, Asian Satellite Campuses Institute, Nagoya University, Nagoya 464-8601, Japan

**Keywords:** mastitis, coagulase-negative staphylococci, *E. coli*, *S. agalactiae*, *S. aureus*, in vitro co-culture

## Abstract

Intramammary infection (IMI) from the environment and infected quarters can cause co-infection. The objective of this study was to determine the ability of coagulase-negative staphylococci (CNS) to survive in the same environment as *Staphylococcus aureus*, *Streptococcus agalactiae*, and *Escherichia coli* as major pathogens. In total, 15 and 242 CNS strains were used in Experiment I and Experiment II, respectively. Both experiments were separated into three conditions: culture with CNS 24 h before (PRIOR), after (AFTER), and at the same time (EQUAL). The lack of a clear zone, regardless of size, was determined to be the key to the survival of both. The CNS species’ percentages of survival against major pathogens were tested using Fisher’s exact test. Differences in the percentages of survival were evident among the CNS species in all conditions. For the PRIOR condition, all CNS mostly survived when living with major strains; however, *S. chromogenes* could degrade *S. agalactiae*. Although most CNS strains were degraded in the AFTER and EQUAL conditions, some strains of *S. hominis* and *S. simulans* could resist *S. aureus* and *S. agalactiae.* In conclusion, some specific strains of CNS are able to survive in an environment with major pathogens. Research into the survival strains may indicate that the concept of novel bacteria with bacteriolytic capabilities might be possible as a novel mastitis treatment.

## 1. Introduction

In 2020, the World Health Organization released a fact sheet indicating that antibiotic resistance is rising to dangerously high levels in all parts of the world, leading to higher medical costs, prolonged hospital stays, and increased mortality [1]. In countries without standard treatment guidelines, antimicrobials are often over-prescribed by health workers and veterinarians, leading to their over-use by the public. The world urgently needs to change the way that it prescribes and uses antimicrobials. Globally, apart from the substantial economic decrease due to disease, bovine mastitis has been associated with the increasing development and rapid emergence of antibiotic-resistant bacteria [2,3]. The recent protocol for antimicrobials used for mild clinical mastitis is recommended to be applied to quarters where the milk samples show specific bacteria growth after obtaining the results of 24 h rapid cultures (for a review, see [4]).

In the context of bovine mastitis, major mastitis pathogens, such as *Staphylococcus aureus*, *Streptococcus uberis*, *Streptococcus agalactiae*, and coliforms, are usually considered more virulent and damaging to the udder than minor mastitis pathogens, such as *Corynebacterium* spp. and coagulase-negative staphylococci (CNS). Spontaneous cure refers to the curing of a disease without any external treatment, whereby the bacteria are destroyed by the udder’s defense mechanism recruitment [5]. The spontaneous curing of mastitis, mostly referring to transient or non-persistent infection, is highly dependent on the identified bacteria. For example, *S. agalactiae* was found to be related to persistent infection [6], while *S. uberis* could be both persistent and transient based on strain [7] and virulence factors [8]. Among the mastitis pathogens, coagulase-negative staphylococci (CNS), categorized as minor pathogens that typically cause subclinical mastitis (SCM), are commonly eliminated by the udder’s immune response within a few days [9,10] without any changes in terms of milk yield and composition [11,12].

Poor mastitis control programs favor the IMI from heterogeneous bacteria, always causing mixed and co-infection [13], and CNS was the most common bacteria for mixed infection [14,15]. Bacteria in the environment and host organisms, for example, in the mammary glands, likely use networks of competitive mechanisms to survive and shape the composition and function of the diverse community [16]. The results of bacterial competition always show a single dominant mastitis pathogen in most mastitis studies [6,17]. In longitudinal mastitis studies in farms with poor mastitis control, further bacterial infections in the same quarter often occurred [7,18]. In addition, Keane et al. [19] demonstrated that the traditional culture methods mostly showed a single dominant pathogen that was the same when identified by PCR. However, the PCR showed extra mixed infections because real-time PCR can detect dead bacterial DNA 20 weeks after inactivation [20].

Evidence that appears to be beneficial for mastitis cows would be the survival of CNS or transient bacteria after co-infection with major or persistent bacteria, subsequently increasing the recruitment of white blood cells and causing a natural, spontaneous cure, as shown in the previous studies [7,18]. The events and mechanisms behind the competition between pathogens in the mammary niche are neither determined nor clearly understood [21]. Approaches to bacterial competition that rely on the culture of two or more organisms together are emerging as a powerful tool to discover and reimagine the mechanisms of competition between bacteria. Therefore, the objective of this study is to determine the abilities of CNS species when either the existing or attacking pathogen survives when living in the same environment as major mastitis pathogens, including *S. aureus, S. agalactiae*, and *E. coli*.

## 2. Results

**Figure 1:**
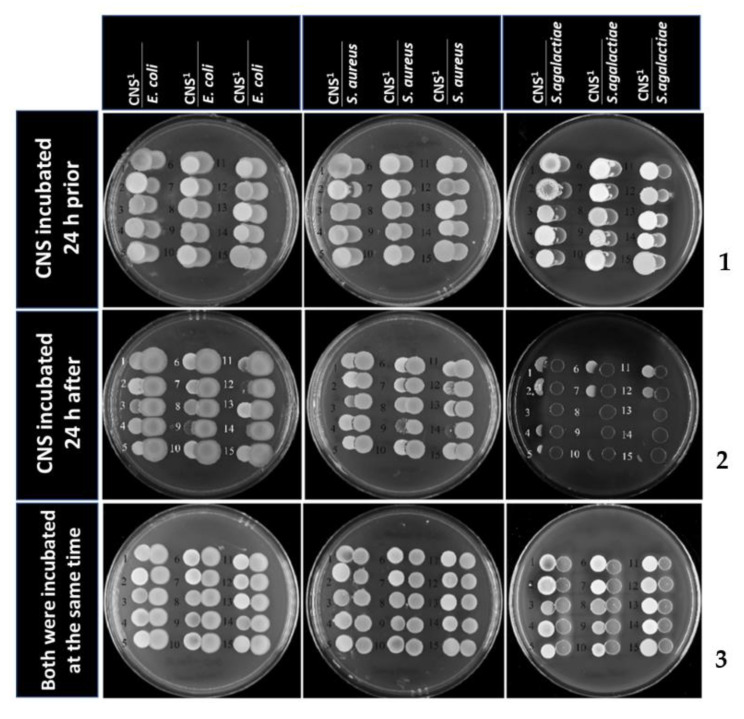
The inhibition zone of major strains toward minor strains in PRIOR condition. (**1**) 24-h prior incubation with CNS (upper), AFTER condition; (**2**) twenty-four-hour prior incubation with major pathogens (middle), EQUAL condition; (**3**) starting incubation at the same time (lower). ^1^ Coagulase-negative pathogens included 1., 2. *S. chromogenes*; 3., 4. *S. epidermidis*; 5., 6. *S. haemolyticus*; 7. *S. simulans*; 8., 9. *S. warneri*; 10. *S. xylosus*; 11. *S. epidermidis*; 12. *S. hominis*; 13. *S. cohinii*; 14. *S. capitis*; 15. *S. sciuri*.

After being co-cultured for 24 h, the results of Experiment I were separated into three different cultured schemes (shown in Figure 1). For the CNS incubated 24 h prior to the PRIOR condition, the sizes of the colonies formed by CNS were different, with the smallest for *S. capitis* (No. 14). Most CNS colonies have wrinkled surfaces, motile outgrowths, and are larger than that of the *S. aureus* and *S. agalactiae*. When cultured for 24 h prior to the addition of major pathogens (upper figure), all CNS were not lysed or had clear zones. Most *E. coli*, *S. aureus*, or *S. agalactiae* colonies were progressively motile and covered parts of the CNS, especially *E. coli* and *S. aureus*, but were not degraded by CNS. All colonies formed by both CNS and *E. coli* remained intact. When co-cultured with *S. chromogenes* strain No. 2, the colonies formed by *S. aureus* and *S. agalactiae* were progressively degraded, and the underlying cells were lysed. For the second scheme, all *E. coli* colonies were larger than CNS, and some of them were progressively motile, degrading the CNS and lysing *S. warneri* (No. 9), *S. hominis* (No. 12), and *S. capitis* (No. 14). The *S. aureus* colonies cultured for 24 h before CNS were progressively moved and degraded, with most of the CNS starting to be lysed. In this scheme, the *S. agalactiae* colonies were clear and intact, with no movement. However, almost all *S. agalactiae* were aggressively attacked, causing all CNS to be lysed, except *S. epidermidis* (No. 11). For example, *S. epidermidis* (No. 3), *S. warneri* (No. 8 and 9), *S. cohinii* (No. 13), and *S. capitis* (No. 14) were entirely degraded and disappeared. For the last scheme (incubation at the same time), the *E. coli* colonies were larger than CNS, *S. aureus*, and *S. agalactiae*. All colonies formed by CNS co-cultured with *E. coli* and *S. aureus* were intact and not degraded, while the three colonies formed by *S. epidermidis* (No. 3) and *S. warneri* (No. 8 and 9) were degraded and lysed.

**Figure 2:**
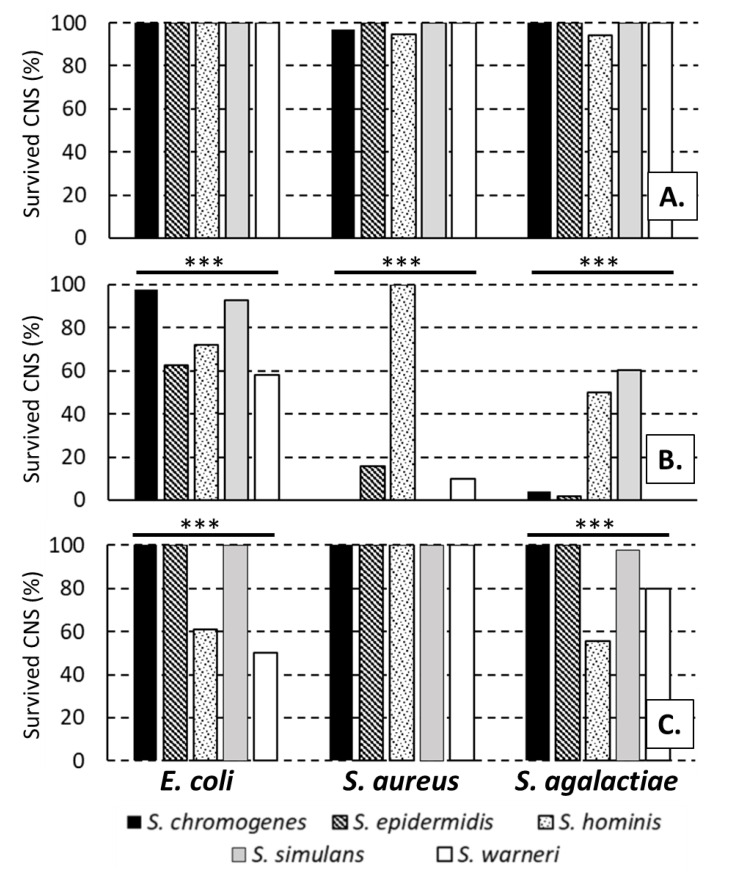
Percentage of the survival ability of CNS strains toward major strains in (**A**) PRIOR condition 1 and 24-h prior incubation with CNS; (**B**) AFTER condition 2 and 24-h prior incubation with major pathogens; (**C**) EQUAL condition 3, starting incubation at the same time. *** Indicated the significant difference in survival rates between species when incubated with the specified major strains at *p* < 0.05.

**Figure 3:**
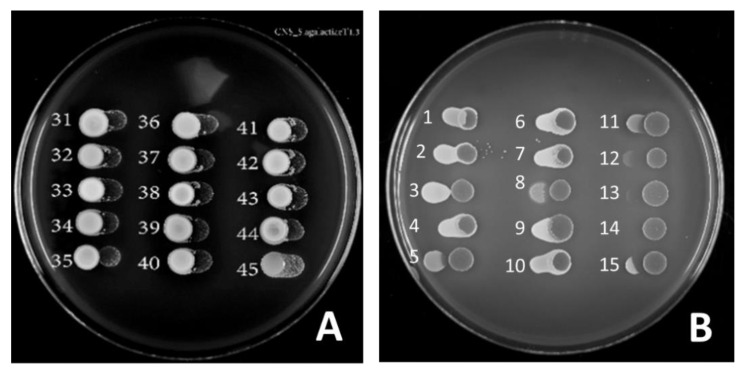
Example of the result of the agar spot competition assays: (**A**) the spots are colonies formed by *S. chromogenes,* the right spots are the colonies formed by *S. agalactiae* (No. 31–45) when the *S. chromogenes* were cultured at 24 h prior (PRIOR), and many colonies formed by *S. agalactiae* are degraded and lysed; (**B**) the spots are colonies formed by *S. hominis*, the right spots are the colonies formed by *S. agalactiae* when the *S. hominis* (No. 1–10) and *S. warneri* (No. 11–15) are cultured at 24 h later (AFTER), and many colonies formed by *S. hominis* (No. 1–4, 6, 7, 9 and 10) are detached and are not degraded by *S. agalactiae*.

Figure 2 shows the interspecies competition in the in vitro mixed intramammary infection situation between major and minor strains (Experiment II). In the PRIOR condition, all field-strain CNS survived with *E. coli*, and almost all field-strain CNS survived with *S. aureus* and *S. agalactiae*, except for very few *S. chromogenes* and *S. hominis* (Figure 2A). In contrast to the other CNS, these can only be survived by *S. agalactiae*, where colonies formed by some *S. chromogenes* demonstrate motile outgrowths. The *S. agalactiae* colonies were degraded and lysed (Figure 3A). When the major pathogens were cultured prior to CNS and for the AFTER condition, the percentages of survival among the species of CNS were significantly different for *E. coli*, *S. aureus*, and *S. agalactiae* (Figure 2B). *S. chromogenes* survived well with *E. coli*; however, most were lysed by *S. aureus* and *S. agalactiae*. In contrast, *S. hominis* had a lower survival rate than *S. chromogenes* and *S. simulans* but could survive well with *S. aureus* and *S. agalactiae* (Figure 2B). Figure 3B indicates the detached colonies formed by *S. hominis* that are not degraded and lysed by *S. agalactiae*. In the EQUAL condition, the percentages of surviving CNS show significant differences when cultured with *E. coli* and *S. agalactiae* (Figure 2C). All CNS survived when cultured at the same time with *S. aureus* for 24 h; however, only *S. chromogenes* and *S. epidermidis* had a 100% survival rate out of the three major pathogens (Figure 2C).

## 3. Discussion

In this study, the agar spot competition assays were used to evaluate the surviving CNS after being with other major bacteria, including *E. coli*, *S. aureus,* and *S. agalactiae*, with the latter two being contagious and persistent [6]. This might represent a unique feature of co-intramammary infections relating to CNS [13]. The designed assays demonstrated a mutual exclusion between the two species depending on the sequence of CNS inoculation at 24 h before, 24 h after, and at the same time as PRIOR, AFTER, and EQUAL, respectively. This imitates the natural IMI that might first infect a cow’s udder with either minor or major pathogens. The CNS species used in this present study were *S. chromogens*, *S. simulans*, *S. epidermidis*, *S. hominis*, and *S. warneri*, which are the most prevalent CNS species isolated from bovine mastitis cases in many countries [22,23].

Regarding the competition between CNS and *E. coli*, both laboratory strains in Experiment I (Figure 1) and all field strains in Experiment II (Figure 2) showed that 100% of the CNS at PRIOR was able to survive after being together; however, some of the *S. hominis* and *S. warneri* did not survive in AFTER and EQUAL conditions. In the PRIOR condition, although the colony formed by *E. coli* could phagocytose the colony formed by all CNS, it could not lyse the colony formed by CNS. Based on our literature review, this is the first report on the evidence of in vitro bacteria competition because this bacterium is more often found in monoculture [24]. Accordingly, our findings might be due to the mild interaction during early co-infection. *Escherichia coli* is a Gram-negative motile bacterium. Therefore, the motility of the *E. coli* strains might play a role in the reduction in the amount of preformed CNS biofilm, which, in turn, might result in the elimination of CNS in a longer infection [25].

Our study found there to be an aggressive degrading of most tested CNS strains when in competition with *S. agalactiae* in the AFTER condition in both Experiments I (Figure 1) and II (Figure 2B). However, *S. epidermidis* and *S. hominis* were able to resist. The strongest inhibitory effect for *S. agalactiae* found in this present study is supported by the studies by Wescombe and Tagg [26] and Karaya et al. [27], who reported that both agalacticin and nisin P. bacteriocins produced by *S. agalactiae* were able to inhibit *Staphylococcus* spp. The inhibitor acts as a self-toxin bacteriocin that kills other bacteria directly observed in major bacteria, especially *S. agalactiae* [28,29].

For the competition with *S. aureus*, most of the results using CNS laboratory strains in Experimental 1 (Figure 1) showed the degraded colony AFTER. Similarly, all CNS field strains in Experiment II (Figure 2) could not resist *S. aureus* AFTER, except *S. hominis*. The repression of *S. aureus* is consistent with the study by Mahmmod et al. [30], who reported the co-habitual competition between *S. aureus* and CNS species within the same niche. This study also supports our findings for the EQUAL condition, in which both species can survive being together.

Overall, most colonies formed by CNSs were not lysed, regardless of the presence of major pathogen species for the PRIOR and EQUAL conditions. This finding might be explained by the release of antimicrobials from the major pathogens being delayed until an achieved local quorum, ensuring the presence of sufficient cell numbers and the production of fully inhibitory antimicrobial levels in the prevailing diffusion environment. Consequently, the AFTER condition, where the population level of the major pathogens reached a critical level, is where a quorum was achieved, resulting in the production and release of antimicrobials [31]. Although most of the CNS strains were progressively degraded by the major pathogens, *S. chromogenes* was the only species that could evade the motile outgrowths and degraded colonies formed by *S. aureus* and *S. agalactiae* (*S. chromogenes* No. 2; Figure 1 and Figure 3A). Moreover, our results also reported a 100% survival rate when growing with all three major pathogens (Figure 2C). This result is consistent with the work of Braem et al. [32], who reported the strongest inhibitory effect of *S. chromogenes* against a wide range of mastitis-causing pathogens and other CNS. Additionally, there are also several mechanisms of *S. chromogenes* that inhibit the growth of major pathogens [33,34,35]. Bacteriocin production is a strain- not species-specific, trait [36]. The *S. chromogenes* strain has the ability to synthesize and secrete bacteriocins and 6-thioguanine (6-TG), a purine analog that suppresses *S. aureus* growth by inhibiting de novo purine biosynthesis. Together with an increase in SCC by *S. chromogenes* strains, which was shown to have elicited udder immune responses, this may offer cross-protection against other mastitis pathogens [37,38], indicating that *S. chromogenes* strains display the strongest inhibitory effects against major mastitis pathogens. In conclusion, both species- and strain-specific CNS are able to survive in an environment with major pathogens. All CNS cultured 24 h before could survive with all major pathogens. Following a 24 h culture, *S. chromogenes* with the PRIOR condition could degrade *S. agalactiae* colonies. When cultured for 24 h after the major pathogens were added, most *S. hominis* survived with *S. aureus*, but only some strains could survive with *E. coli* and *S. agalactiae*.

## 4. Conclusions

Based on the results of this study, *S. chromogenes and S. hominis* play a role in the natural, spontaneous curing of mastitis from *S. aureus* and *S. agalactiae*, both major and persistent pathogens, by either destroying the infection or surviving in the udder to accelerate the udder’s defense mechanism. This indicates that the concept of novel bacteria with bacteriolytic capabilities might be possible as a novel mastitis treatment. However, further studies are needed: (i) the evaluation of the ability to induce white blood cells into an infected site for pathogen clearance, (ii) the elucidation of the mode of action against major pathogens, (iii) an evaluation of the procedure’s safety regarding the cytotoxicity of the lived bacteria intramammary infection assessed by in vitro and in vivo tests, and (iv) an evaluation of the long-term effects of using novel bacteria with bacteriolytic capabilities.

## 5. Materials and Methods

### 5.1. Bacterial Strains and Study Design

Two experiments were conducted to determine the survival of minor pathogens in an environment with major pathogens. For Experiment I, 15 isolates of various CNS species from mastitis samples, ATCC, and the Microbiology Laboratory in the Faculty of Science, Chiangmai University, were used (Table 1). For Experiment II, all strains of CNS species stocked in our database, and the randomly selected *Streptococcus agalactiae* used in this study were obtained from frozen mastitis pathogen stock (−80 °C) from the Laboratory of Milk Quality and Mastitis, the Faculty of Veterinary Medicine, Chiangmai University. The stocked bacteria originated from milk samples of subclinical mastitis from lactating cows in smallholder dairy farms in different areas of Thailand, including northern (n = 131), northeastern (n = 75), and central (n = 36) regions. From the total of 242 stocked CNS strains, 5 staphylococci species, including *S. chromogens* (n = 136), *S. simulans* (n = 43), *S. epidermidis* (n = 32), *S. hominis* (n = 18), and *S. warneri* (n = 13) were used. All stocked strains were identified by conventional bacterial culture [39] and confirmed by Maldi-Tof. The three main major pathogens were *Staphylococcus aureus* ATCC25923, *E. coli* ATCC25922, and the field strain of *Streptococcus agalactiae*, as previously described. All strains were recovered in Tryptic Soya broth (TSB) (Himedia, Mumbai, India) and grown overnight at 37 °C. After that, the inoculums were cultured on 5% bovine blood agar (HIMEDIA, Mumbai, India) at 37 °C for 24 h.

To imitate a mixed infection situation in a mammary gland, all CNS strains were separately cultured with the selected major pathogens in one of three conditions: (1) minor pathogens were inoculated first and allowed to grow overnight before each major pathogen was inoculated nearby, which the intramammary was the first to be infected with CNS (PRIOR); (2) vice versa, where the first was infected with the major pathogens followed by the CNS (AFTER); (3) both bacteria inoculated at the same time as if the infection happened at the same time in the udder (EQUAL). The first inoculated bacteria were defined as the early colonizer, and the following inoculated bacteria were defined as the later colonizer.

### 5.2. Agar Spot Competition Assays

Agar spot competition assays were used in both experiments. The protocol was applied according to what was previously described by Kaspar et al. [40] with modifications. Briefly, the bacterial suspension was adjusted to a 0.5 McFarland turbidity standard to achieve 10^8^ CFU/mL from single overnight colonies. Five µL of bacterial suspension was inoculated onto tryptic soy agar (TSA) (HIMEDIA, Mumbai, India) as the early colonizer. After overnight incubation at 37 °C, 5 µL of the competing strains from another group at the same concentration were inoculated beside the early colonizer as the later colonizer. The plates were further aerobically incubated at 37 °C overnight, then the cleared zone between colonies in three different experimental conditions was observed. This experiment was repeated twice, with two independent biological replicates in each experiment.

### 5.3. Statistical Analysis

For Experiment I, the data were described using pictures of the result plates. For Experiment II, the presence of a clear zone between the culture spots was used to measure the interbacterial competition between major and minor strains. Data on the presence or absence of an inhibition zone around CNS inoculation were determined as negative and positive interactions, respectively. Negative interaction was described when a zone of inhibition was present around a major strain, and no visible growth for the minor strains was found within the same area. However, if there was no inhibition zone or the resistant minor strains were able to grow within the zone of inhibition, this was interpreted as positive interactions. Percentages of survival ability of each CNS species were defined by the number of specified CNS species with the positive interaction divided by the total number of the specified CNS species. The percentage differences in the survival ability of each CNS species among the different major strains were tested using Fisher’s exact test (the SAS University Edition). The significance levels were set at *p* < 0.05, and tendencies were set at *p* < 0.1.

## Figures and Tables

**Table 1:** Source of coagulase-negative staphylococci (CNS) as minor mastitis pathogens used in Experiment I.

No.	Species	Source
1, 2	*Staphylococcus chromogenes*	Mastitis quarter
3, 4	*Staphylococcus epidermidis*	Mastitis quarter
5, 6	*Staphylococcus haemolyticus*	Mastitis quarter
7	*Staphylococcus simulans*	Mastitis quarter
8, 9	*Staphylococcus warneri*	Mastitis quarter
10	*Staphylococcus xylosus*	Mastitis quarter
11	*Staphylococcus epidermidis*	ATCC
12	*Staphylococcus hominis*	Faculty of Science
13	*Staphylococcus cohinii*	Faculty of Science
14	*Staphylococcus capitis*	Faculty of Science
15	*Staphylococcus sciuri*	Faculty of Science

## Data Availability

All data are shown in the manuscript.
